# Should Poor Law Infirmaries Have a Visiting Medical Staff?

**Published:** 1909-10-23

**Authors:** F. S. Toogood

**Affiliations:** Barrister-at-Law, Medical Superintendent of Lewisham Infirmary


### SHOULD POOR LAW INFIRMARIES HAVE A VISITING MEDICAL STAFF?

By F. S. TOOGOOD, M.D.Lond., D.P.H., Barrister-at-Law, Medical Superintendent of Lewisham Infirmary.

The question is not by any means new, and might fitly be described as a chronic interrogation, varied by acute exacerbations. It has certainly been discussed since the separation of the infirmaries from the workhouses in the 'seventies.

The query carries the suggestion that the proper way of supplying medical treatment to the occupants of an institution for the treatment of the sick is by means of a visiting staff. The method is that which is adopted generally in this country by the Voluntary hospitals, and it is agreeable to these institutions, inasmuch as it supplies them with the services of trained physicians and surgeons who ask for no remuneration, while the posts are eagerly sought for by members of the profession, who see in them the means for the rapid acquisition of experience and consequently of publicity, practice, pecuniary benefits and numerous social advantages. The voluntary hospital system, having been in existence centuries before the advent of the infirmary, has inevitably become the standard, and institutions conducted in a different method have necessarily to justify their departure from convention. When the sick wards formed part of the workhouse, the medical officer was of necessity a general practitioner who devoted a few hours daily to the treatment of the sick inmates. With the development of the idea of treating the necessitous sick in institutions, the increase of work was met by the appointment of a resident medical officer working under the visiting doctor. The administrative anomalies involved in the position of the sick in the workhouse and the consequently subordinate position of the medical staff and nurses to the workhouse authorities, led to the separation of the sick wards from the control of the workhouse and to the erection of the separate infirmary. The appointment of medical officers rested with the local authorities, and although the duties involved the whole time of those elected, yet the salaries were invariably small, and the choice of candidates was limited. Add to this the fact that the medical education of the ordinary practitioner 40 years ago was not what it is now, and the relatively slow development of the infirmaries during the first fifteen or twenty years of their existence is easily understood.

The next generation of medical officers succeeded to a different condition of affairs. After years of fighting the position of the medical superintendent as the responsible head of the institution was secure; the comfort and kindly treatment found in the infirmaries had removed much of the prejudice justly entertained against the old workhouse wards, and the stigma of pauperism was insensibly but surely fading from those who claimed the help of the community because of their sickness. Every institution gets the class of patient which it deserves, and those which deserve the best patients get them. For many years the nursing of infirmaries was a very weak point, the matrons were untrained women, usually excellent housekeepers, but quite ignorant of nursing details. It was therefore impossible to get the best class of charge nurses or sisters, while the assistant nurses were eminently unsatisfactory. It was seen that the only hope of salvation lay in the conversion of each infirmary into a training school for nurses, and luckily, at that time, nursing was a fashionable craze, so little difficulty was experienced in effecting the alteration, which has succeeded admirably. Hospital-trained sisters are obtained without difficulty for the higher posts, and to-day there is not an untrained matron in London.

The necessity for giving the nurses a full and thorough training justified medical officers keen upon surgery in pursuing their predilection, while it compelled those less surgically inclined to cultivate the requisite knowledge. The local authorities varied in their attitude, some being wholly enthusiastic, others frankly antagonistic. Whitehall, which naturally has a wider view of the subject and has to take into account possible social and economic results, did not throw any obstacles into the way of progress, but "had not contemplated the conversion of infirmaries into schools for surgery." The point to which we have now reached is that there are 26 separate infirmaries in London containing 14,863 beds, employing 1,756 nurses, and that 3,894 operations under anæsthetics are performed yearly. The number of medical officers varies with the number of beds in the infirmary, and also with the number of institutions to which their services extend. In some there is a medical superintendent and one assistant medical officer, in most there are two assistants, and in some three, four or five.

One infirmary contains 284 beds, another as many as 840. Local conditions vary greatly, and with them the type of patient and the variety of disease. In districts adjacent to a general hospital the infirmary will contain very few acute medical cases, and practically no surgical patients at all; indeed the wards will be occupied almost entirely by persons suffering from senile decay, and by those who have been refused admission to or have been discharged from general hospitals because of the chronic character of their diseases. On the other hand, there are regions in London relatively remote from a hospital of repute, and here the class of patient depends entirely upon the character of the work attempted in the infirmary. At Lewisham, some parts of which are ten miles from Guy's Hospital, the number of acute and surgical cases has been relatively large ever since its opening in 1874; with a working number of 369 beds, there were admitted last year 3,376 cases, a number which is actually greater than that shown by other infirmaries having twice the quantity of beds. Nearly 600 operations under anæsthetics are performed yearly, including complete hysterectomies, gastro-jejunostomies, and intestinal anastomoses, all perfectly successful. In 15 years I have operated 38 times for ruptured gastric ulcer, with 32 recoveries. Naturally the work is hard, there being also the workhouse, the scattered Homes for Children, and the infectious wards to attend. It is not generally known that measles, German measles, whooping cough, chicken pox, erysipelas, and puerperal fever are not admitted to the hospitals of the Metropolitan Asylums Board, and therefore have to be provided for by the Poor Law Guardians. Up to the present time all the work coming to us has been dealt with in an honest and workmanlike manner—nothing has been turned away or shirked. So far as the infirmary is concerned the medical staff is quite capable of performing all the work which has hitherto presented itself.

The appointment of a visiting staff must be either in addition to or in place of the medical superintendent.

The first alternative I hold to be entirely unnecessary if care be taken to appoint capable men as medical officers. The latter plan would involve a complete reversal of the policy, for which the medical profession has been striving for years—namely, that the medical officer shall be in full control and solely responsible to the governing body for all matters. The experiment has been tried in one instance, and has resulted, I am informed by former assistant medical officers, in an uncomfortable condition of affairs. There is a lay superintendent, a matron, two visiting medical officers, and two or more resident assistant medical officers. The elevation of a layman over the professional element in an institution for the sick is retrograde and unworkable, because the opportunities for friction are so numerous that, unless all parties are endowed with exceptional tact, unpleasant squabbles are sure to occur. The visiting medical officers having greater interests outside have a distinct inducement to leave the treatment of the chronic and less interesting cases to the junior and less experienced medical officers, confining their attention to those patients who present interesting features. The numerous points in medical and institutional administration which are ever arising must be inadequately dealt with. The visiting staff have neither the knowledge, the experience, nor the point of view to enable them to cope with such questions. The resident medical officers have neither the *savoir faire* nor the authority which would command success in such negotiations. Hence there will always be the incessant turmoil, bickering, and institutional inefficiency which invariably have ensued under similar conditions.

The interests of the governing body run upon two lines. The first to pay salaries which do not err upon the side of generosity. They are therefore unlikely to pay for a trained medical administrator and also for a visiting staff. Secondly, for administrative reasons they must have a responsible officer whom they can trust, to whom they look for guidance, and whom they call to account for the efficiency of the institution in every detail. The actual treatment of patients is only one of the many duties of which a superintendent must have a complete acquaintance. The question of the salaries to be paid to a possible visiting staff needs very careful consideration. An infirmary is a State hospital, and is not in any sense a charity; consequently, there is no justification for the services of a staff being obtained either gratuitously or at a low rate of payment. Such service would result in an additional tax being placed upon the medical profession for the benefit of the community at large. I am informed that in a certain Midland town the services of a surgeon and of a physician have been obtained at a salary of about £100 each per annum. By a confusion of ideas they are performing services for the State as if they were acting for a charity. The State has no right to their services except in return for an adequate and sufficient payment at the standard rate.

In conclusion, I am of opinion that much can be done to remedy any local inadequacy which may exist. The present union as the unit of administration is too small. I would make London into one area. Classification of various diseases and the specialising of medical officers would then be easy. I look for the creation of a State Civil Medical Service with adequate payment for its members. I should regard the appointment of a visiting staff as a retrograde step, bringing no advantage to the sick, placing an additional burden upon an already overtaxed profession, and delaying the formation of that State Medical Service, which I regard as essential to the solution of many problems.

